# Femoral neck system vs. cannulated screws on treating femoral neck fracture: a meta-analysis and system review

**DOI:** 10.3389/fsurg.2023.1224559

**Published:** 2023-07-18

**Authors:** Yimin Zhou, Zongyang Li, Kecheng Lao, Zixiu Wang, Li Zhang, Shiyou Dai, Xiao Fan

**Affiliations:** ^1^ College of Traditional Chinese Medicine, Changchun University of Chinese Medicine, Changchun, China; ^2^ Department of Osteoarticular and Sports Medicine, Qingdao Municipal Hospital, Qingdao, China; ^3^ College of Pharmacy, Gannan Medical University, Ganzhou, China; ^4^ Department of Rehabilitation and Health, Fujian Vocational College of Bio-engineering, Fuzhou, China

**Keywords:** femoral neck fractures, femoral neck system, cannulated compression screws, meta-analysis, system review

## Abstract

**Objective:**

This meta-analysis aimed to compare the relative safety and efficacy of cannulated compression screw (CCS) and femoral neck system (FNS) in treating patients with femoral neck fractures and to provide evidence-based medical evidence for FNS in treating femoral neck fractures.

**Methods:**

PubMed, Embase, Cochrane, and China National Knowledge Infrastructure databases were searched to collect outcomes related to femoral neck fractures treated with FNS and CCS, including time to fracture healing, incidence of non-union, incidence of osteonecrosis of the femoral head, incidence of failure of internal fixation, rate of femoral neck shortening, Harris hip score, Barthel index, operative time, intraoperative blood loss, fluoroscopy frequency, and complications. A meta-analysis was performed using RevManv5.4 (The Cochrane Collaboration) and Stata v14.0 software.

**Results:**

This analysis included 21 studies involving 1,347 patients. The results showed that FNS was superior to CCS in terms of fracture healing time [mean difference (MD) = −0.75, 95% CI = (−1.04, −0.46), *P *< 0.05], incidence of bone non-union [odds ratio (OR) = 0.53, 95% CI = (0.29, 0.98), *P *= 0.04], incidence of osteonecrosis of the femoral head [OR = 0.49, 95% CI = (0.28, 0.86), *P *= 0.01], incidence of internal fixation failure [OR = 0.30, 95% CI = (0.18, 0.52), *P* < 0.05], rate of femoral neck shortening [OR = 0.38, 95% CI = (0.27, 0.54), *P *> 0.05], Harris hip score [MD = 3.31, 95% CI = (1.99, 4.63), *P *< 0.001], Barthel index [MD =  4.31, 95% CI = (3.02, 5.61), *P *< 0.05], intraoperative bleeding [MD = 14.72, 95% CI = (8.52, 20.92), *P *< 0.05], fluoroscopy frequency [OR = 0.53, 95% CI = (0.29, 0.98), *P *= 0.04], and complications [OR = 0.31, 95% CI = (0.22, 0.45), *P *< 0.05]. The difference between FNS and CCS in operative time was not statistically significant [MD = −2.41, 95% CI = (−6.88, 2.05), *P *= 0.29].

**Conclusion:**

FNS treatment of femoral neck fracture can shorten the fracture healing time; reduce the incidence and translucent rate of bone non-union, osteonecrosis of the femoral head, and internal fixation failure; reduce intraoperative blood loss and postoperative complications; and improve hip joint function and activity. We are confident in the findings that FNS, an effective and safe procedure for internal fixation of femoral neck fractures, is superior to CCS.

## Introduction

A femoral neck fracture is a common clinical lower limb fracture, accounting for 48%–54% of hip fractures (1). In elderly patients, the injury factor is primarily low-energy injury. However, the injury factors for young and middle-aged patients are mostly violent injuries, such as car accidents and high-altitude fall injuries. Due to the characteristics of the vascular anatomy of the femoral neck, femoral neck fractures often lead to complications, such as avascular necrosis of the femoral head and non-union of fractures. Therefore, how to effectively treat femoral neck fractures has always been a clinical problem. Currently, internal fixation surgery is one of the key methods for treating femoral neck fractures. Internal fixation surgery is preferred, especially for young and middle-aged patients with femoral neck fractures. The internal fixation methods commonly used in clinical practice for treating femoral neck fractures include cannulated compression screw (CCS), dynamic hip screw, and medial steel plate combined with CCS. Among them, CCS has the most extensive clinical application. Although CCS achieves certain efficacy in treating femoral neck fracture, it often leads to complications such as osteonecrosis of the femoral head, non-union of fracture, hip varus, and failure of internal fixation. Therefore, the internal fixation of femoral neck fracture is still a research hotspot in recent years. Over the past few years, DePuy Synthes has developed a new internal fixation system for fixing femoral neck fractures, the femoral neck system (FNS), which is considered to have the advantages of short operation time, minor trauma, mechanical stability, sliding compression, and minimally invasive implantation. However, the advantages of FNS in the treatment of femoral neck fracture compared with traditional CCS are still controversial, its clinical application time is still short, and there is a lack of evidence-based medical evidence on its efficacy and safety in treating femoral neck fracture. Therefore, this study conducted a meta-analysis and systematic review of the clinical efficacy of FNS and CCS in the treatment of femoral neck fracture; clarified the advantages, disadvantages, and safety of each method in the treatment of femoral neck fracture; and provided evidence-based medical evidence for FNS in the treatment of femoral neck fracture, guiding its clinical application.

## Materials and methods

### Search strategy

The PubMed, Embase, Cochrane, and China National Knowledge Infrastructure databases were searched until 8 November 2022. Keywords included (1) “femoral neck system” and “FNS”; (2) “cannulated screws,” “cannulate compression screw,” “CCS,” and “CS”; and (3) “femoral neck fracture” and “FNF.” Supplementary Tables S1–S3 describe the search strategies.

#### Eligibility criteria

The inclusion criteria include (1) patients: adults with the first diagnosis of unilateral femoral neck fracture; (2) intervention: FNS-fixed experimental group; (3) control: CCS-fixed control group; (4) results: fracture healing time, rate of bone non-union, rate of osteonecrosis of the femoral head, rate of internal fixation failure, Harris hip joint score, Barthel index, operation time, intraoperative blood loss, the dialysis rate, and complications; and (5) study design: prospective cohort studies, retrospective comparative controlled trials, and randomized controlled trials (RCT).

The exclusion criteria include (1) review articles, conference summaries, comments, and biomechanical studies and (2) patients with pathological or open femoral neck fractures.

### Study selection

Two investigators (YZ and XF) independently screened the articles according to the eligibility criteria (2). For the review articles, the relevant reference articles were screened. Two investigators (YZ and XF) independently performed a preliminary screening of each article based on the title and abstract and then read through the full text for further screening. Any discrepancies were resolved by consensus with a third investigator (ZL).

### Data extraction and quality assessment

Two researchers independently extracted data from the identified articles using the standardized form. The extracted data included the first author, year of publication, type of study, age, sex ratio, affected side, Garden classification, Pauwels classification, cause of injury (fall, traffic accident, or higher fall), follow-up time, and outcome measures. If there were inconsistencies in data extraction among investigators, a consensus was reached through discussion. ROBINS-I was used to assess the risk of bias in non-randomized clinical studies (3), and the Cochrane Collaboration risk-of-bias tool was used in RCT trials (4).

### Statistical analysis

Data were analyzed using RevManv5.4 (The Cochrane Collaboration) and Stata v14.0 software. Odds ratio (OR) and mean differences (MD) with 95% confidence intervals (CI) were used to assess categorical and continuous variables, respectively. Cochran's *Q* and *I*^2^ tests were used to determine heterogeneity. When *I*^2^ was greater than 50% or the *P*-value of the *Q* statistic was less than 0.05, the random-effects model was used; otherwise, the fixed-effects model was used. The funnel chart and Egger's test were used to investigate publication bias (5).

## Results

### Search results

**Figure 1:**
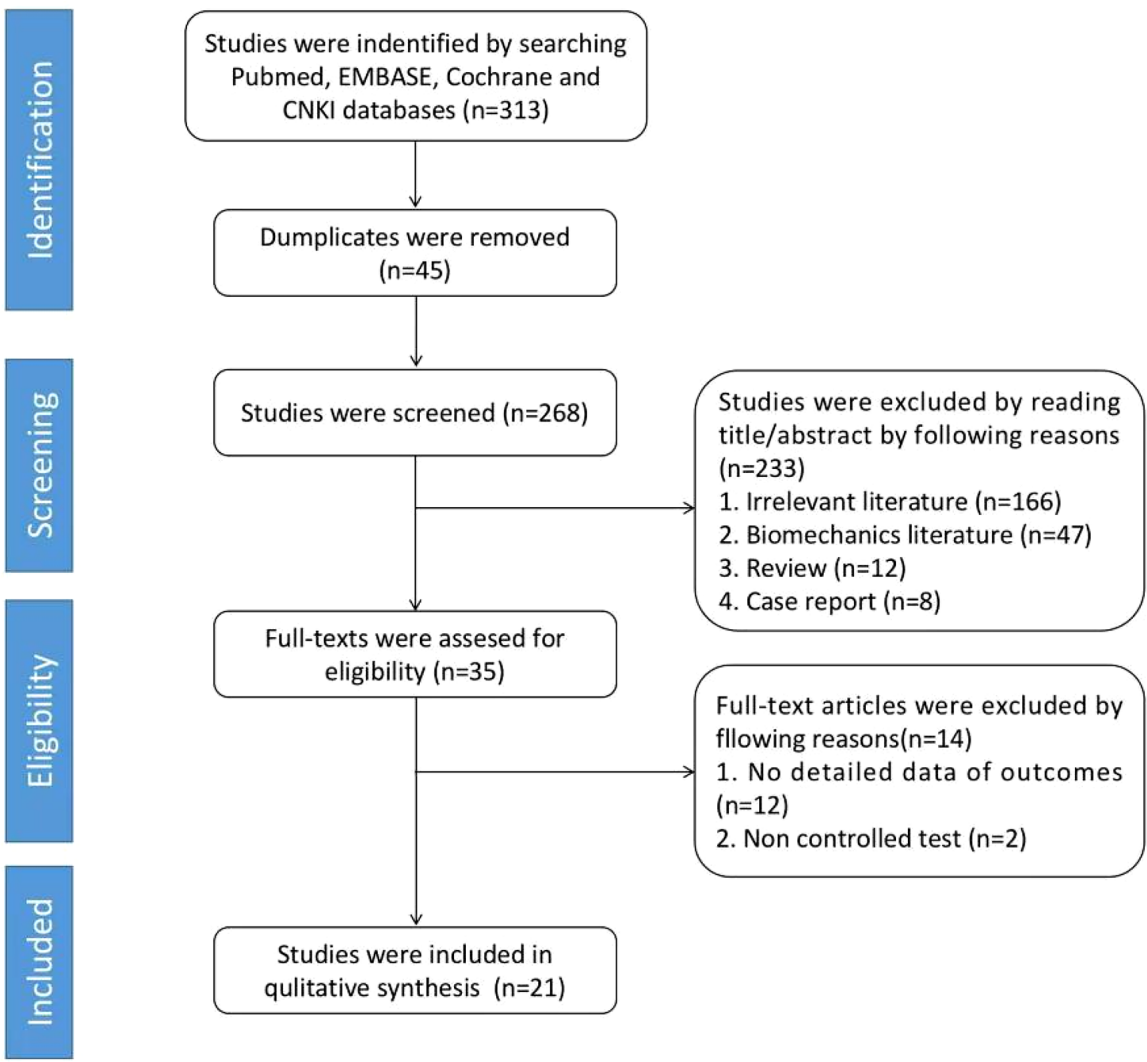
Flow chart of the document retrieval process.

Figure 1 depicts the flow chart of article selection. A total of 313 articles were identified through the electronic database search, of which 45 were preliminarily screened and excluded and 268 were retained. Through the screening of titles and abstracts, 233 articles were excluded, including relevant literature (*n* = 166), biomechanical literature (*n* = 47), review (*n* = 12), and case report (*n* = 8), resulting in 35 qualified literature. A total of fourteen studies were further excluded for the following reasons: no detailed outcome data (*n* = 12) and uncontrolled trials (*n* = 2). Finally, a total of 21 studies were included for further review (6–26) (Figure 1).

### Characteristics of the included studies

The 21 studies included in the review were all retrospective cohort studies, of which 20 were conducted in China and 1 was in Switzerland, involving 1,347 patients in total. There were 612 cases in the FNS group (356 males and 256 females) and 735 cases in the CCS group (436 males and 299 females). There were no significant differences between the two groups in terms of age (6–26), sex (6–26), affected side (8, 10, 13–17, 19, 22–25), Garden classification (6–9, 12, 13, 15–22, 24, 25), Pauwels classification (6, 8, 10, 18, 20–22), fracture to treatment time (10, 12, 14, 16, 17, 19–22, 24), and cause of fracture (7, 9, 10, 13, 15–18, 20–22, 25). The follow-up time of the included studies ranged from 3 to 24 months. A total of 16 studies analyzed the fracture healing time, 16 studies reported the incidence of non-union, 20 studies reported the incidence of osteonecrosis of the femoral head, 20 studies analyzed the incidence of internal fixation failure, 18 studies reported the Harris hip joint score, 3 studies analyzed the Barthel index, 20 studies recorded the operative time, 15 studies recorded the intraoperative blood loss, 16 studies reported fluoroscopy frequency, and 18 studies analyzed the complications. Table 1 shows the basic characteristics of the included studies.

**Table 1:** Characteristics of 21 included studies.

Study	Group	*n*, M/F	Left/right	Age, years	Garden classification (I/II/III/IV)	Pauwels type (I/II/III)	Cause of injury, slide/TA/FFH	From injury to surgery, days	Follow-up, months	Outcomes
Chang et al. (20)	FNS CCS	29,14/15 29,14/15	NR	48.2 ± 12.0 48.2 ± 12.3	0/10/4/15 0/12/4/13	6/6/17 6/7/16	28/0/1 26/2/1	4.7 ± 2.2 5.0 ± 2.8	≥6 ≥6	①②③④⑤⑥⑦⑧⑪
Ge et al. (23)	FNS CCS	44,35/9 35,29/6	30/14 19/16	44.7 45.9	NR	NR	18/11/15 14/8/13	NR	3 3	①②③④⑤⑥⑧⑨⑩
He et al. (7)	FNS CCS	33,18/15 36,22/14	NR	50.61 ± 10.30 47.58 ± 10.31	1/8/19/5 2/9/20/5	NR	21/12/- 25/11/-	NR	16.91 ± 3.01 16.91 ± 3.01	①②③④⑤⑥⑧⑩⑪
He et al. (21)	FNS CCS	36,21/15 40,24/16	NR	48.7 ± 8.3 50.2 ± 8.1	0/13/15/8 0/12/19/9	7/13/16 9/18/13	21/15/- 22/18/-	2.7 ± 1.0 2.6 ± 0.8	15.2 ± 1.7 15.2 ± 1.7	①②③④⑤⑥⑧⑩⑪
Hu et al. (6)	FNS CCS	20,12/8 24,14/10	NR	50.45 ± 8.45 50.46 ± 9.26	0/6/8/6 4/6/7/7	1/14/5 4/13/7	NR	NR	6 6	①②③④⑤⑥⑧⑨⑪
Lin et al. (9)	FNS CCS	27,7/20 31,9/22	NR	57.9 ± 7.1 57.6 ± 8.7	0/5/15/7 0/6/17/8	NR	17/4/6 16/5/6	NR	≥13 ≥13	①②③④⑤⑥⑧⑨⑪
Vazquez et al. (11)	FNS TS (CCS)	15,13/2 32,28/4	NR	86.1 ± 4.6 85.0 ± 6.6	NR	NR	NR	NR	3–16 3–16	⑧⑪
Lu et al. (10)	FNS CCS	28,19/9 30,22/8	16/12 13/17	14.5 ± 1.6 14.3 ± 1.5	NR	2/18/8 8/13/9	8/14/6 6/10/14	3.2 ± 1.5 3.6 ± 1.9	16.3 ± 2.0 17.0 ± 2.5	①②③④⑤⑧⑨⑩⑪
Ren et al. (13)	FNS CCS	32,16/16 38,19/19	13/19 13/25	49.4 ± 11.0 48.8 ± 10.1	0/10/12/10 0/12/15/11	NR	22/10/- 27/11/-	NR	11.5 ± 2.9 11.7 ± 3.4	①②③④⑤⑥⑦⑧⑨⑪
Tang et al. (8)	FNS ICCS	47,34/13 45,37/8	26/21 23/22	57.4 ± 15.0 54.8 ± 11.7	0/6/29/12 5/5/31/9	5/12/30 6/10/29	NR	NR	14–24 14–24	①②③④⑤⑥⑧⑨⑩
Wang et al. (25)	FNS CCS	14,10/4 10,6/4	9/5 6/4	41.23 ± 3.87 42.04 ± 5.20	4/3/6/1 1/3/5/1	NR	-/5/6 -/3/6	NR	6–20 6–20	①②③④⑤⑥⑧⑨⑩⑪
Xiong et al. (17)	FNS CCS	62,38/24 57,42/15	38/24 42/15	54.0 ± 13.0 53.2 ± 11.3	0/13/34/15 0/9/33/15	NR	41/12/9 36/11/10	1.41 ± 0.55 1.55 ± 0.46	14.6 ± 1.7 15.1 ± 1.6	①②③④⑤⑥⑧⑨⑩⑪
Yan et al. (18)	FNS CCS	24,10/14 58,38/20	NR	52 (47, 63) 49 (47, 56)	0/4/12/8 2/10/32/4	0/6/18 0/22/36	14/6/4 36/6/16	NR	13.6 (1, 18) 7.3 (3, 12)	①②③④⑤⑥⑧⑨⑪
Yang et al. (14)	FNS ITCS	28,17/11 31,17/14	18/10 14/17	51 (45, 56) 49 (39, 51)	0/5/12/11 0/4/16/11	NR	5/17/6 3/20/6	3.8 ± 1.6 4.0 ± 1.2	11.1 ± 3.3 11.4 ± 2.6	③④⑤⑥⑧⑪
Yang et al. (19)	FNS CCS	47,30/17 47,26/21	30/17 22/25	47.8 ± 9.8 43.7 ± 13.1	NR	3/16/28 6/20/21	NR	4.0 4.0	8.7 ± 3.1 9.1 ± 2.7	①③④⑤⑥⑧⑨⑪
Yang et al. (22)	FNS CCS	15,9/6 29,12/7	9/6 12/7	42.0 41.2	1/3/10/1 2/4/11/2	NR	NR	1.11 1.07	6 6	①②③④⑤⑥⑨⑩⑪
Zhang et al. (12)	FNS CCS	31,14/17 34,16/18	15/16 19/15	51.8 ± 12.5 50.4 ± 12.0	4/2/16/9 7/2/22/3	NR	NR	1.91 ± 0.76 1.80 ± 0.64	9.7 ± 3.5 10.1 ± 2.9	③④⑥⑧⑩⑪
Zhang et al. (24)	FNS CCS	33,11/22 36,15/21	NR	57.61 ± 11.87 52.50 ± 10.72	0/10/9/14 0/12/14/10	NR	NR	1.79 ± 0.86 1.56 ± 0.73	6 6	①②③④⑥⑦⑧⑨⑪
Zhao et al. (15)	FNS CCS	11,7/4 20,9/11	5/6 11/9	46.64 ± 16.32 48.90 ± 12.38	0/7/3/1 0/8/10/2	NR	9/2/0 16/2/2	NR	6–16 6–16	①③④⑤⑥⑧⑨⑩⑪
Zhou et al. (26)	FNS CCS	30,12/18 51,22/29	NR	54.53 ± 6.71 53.14 ± 7.19	NR	NR	NR	NR	10–22 10–22	②③④⑥⑧⑨⑪
Zhu et al. (16)	FNS CCS	15,9/6 32,15/17	10/5 18/14	52.5 ± 7.18 52.88 ± 8.49	0/1/6/8 3/6/10/13	NR	7/6/2 23/5/4	3.6	≥6 ≥6	①②③④⑥⑧⑨

CCS, cannulate compression screw; M, male; F, female; FNS, femoral neck system; NR, not reported; TA, traffic accident; FFH, fall from height. Outcomes: ①: healing time; ②: fracture non-union incidence; ③: femoral head necrosis; ④: internal fixation failure rate; ⑤ femoral neck shortening rate; ⑥: Harris hip score; ⑦: Barthel index; ⑧: operative time; ⑨: intraoperative blood loss; ⑩: fluoroscopy frequency; ⑪: complications.

### Quality assessment of the clinical controlled studies

Most of the included studies (6–10, 12–26) were at low to moderate risk of bias. However, only one study (11) potentially had data loss. (Table 2 shows specific methodological quality evaluations of literature.)

**Table 2:** Quality assessment of the clinical controlled studies.

Study	Confounding	Selection of participants	Classification of interventions	Deviations from intended interventions	Missing data	Measurement of outcomes	Selection of reported results	Overall
Chang et al. (20)	Low	Low	Low	Low	Low	Low	Low	Low
Ge et al. (23)	Low	Low	Low	Low	Moderate	Low	Low	Low
He et al. (7)	Low	Low	Low	Low	Moderate	Low	Low	Low
He et al. (21)	Low	Low	Low	Low	Low	Low	Low	Low
Hu et al. (6)	Low	Low	Low	Low	Moderate	Low	Low	Low
Lin et al. (9)	Low	Low	Low	Low	Moderate	Low	Low	Low
Vazquez et al. (11)	Moderate	Moderate	Low	Low	Serious	Low	Low	Moderate
Lu et al. (10)	Low	Low	Low	Low	Low	Low	Low	Low
Ren et al. (13)	Low	Low	Low	Low	Low	Low	Low	Low
Tang et al. (8)	Moderate	Low	Low	Low	Low	Low	Low	Low
Wang et al. (25)	Low	Low	Low	Low	Low	Low	Low	Low
Xiong et al. (17)	Low	Low	Low	Low	Low	Low	Low	Low
Yan et al. (18)	Low	Moderate	Low	Low	Low	Low	Low	Low
Yang et al. (14)	Low	Low	Low	Low	Low	Low	Low	Low
Yang et al. (19)	Low	Low	Low	Low	Low	Low	Low	Low
Yang et al. (22)	Low	Moderate	Low	Low	Low	Low	Low	Low
Zhang et al. (12)	Low	Low	Low	Low	Low	Low	Low	Low
Zhang et al. (24)	Low	Low	Low	Low	Low	Low	Low	Low
Zhao et al. (15)	Low	Moderate	Low	Low	Low	Low	Low	Low
Zhou et al. (26)	Low	Moderate	Low	Low	Moderate	Low	Low	Moderate
Zhu et al. (16)	Low	Moderate	Low	Low	Low	Low	Low	Low

### Results of meta-analysis

The 21 studies included (6–26) analyzed the fracture healing time, incidence of bone non-union, incidence of osteonecrosis of the femoral head, incidence of femoral neck shortening, incidence of internal fixation failure, Harris hip joint score, Barthel index, operation time, intraoperative blood loss, fluoroscopy frequency, and complications of the two fixation methods.

#### Healing time

**Figure 2:**
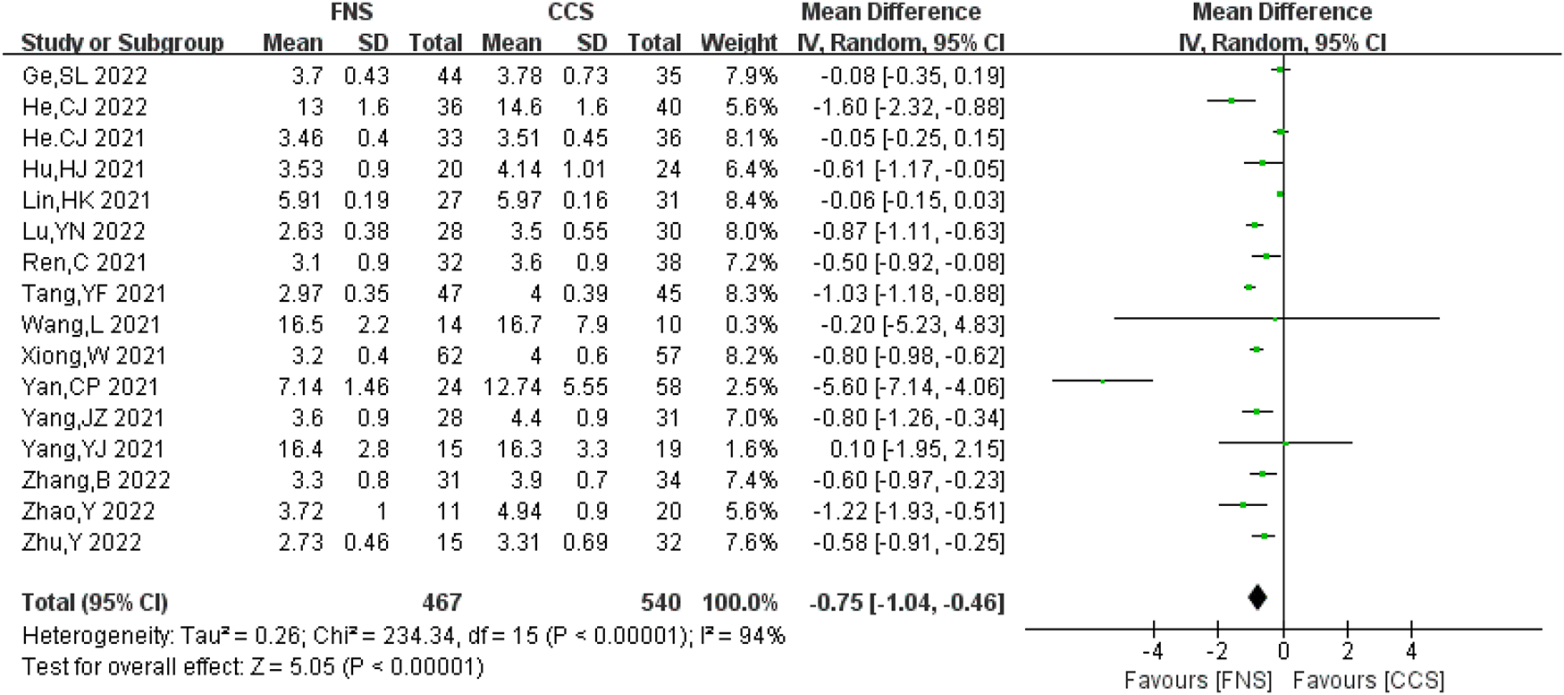
Forest plot comparisons for healing time.

A total of 16 studies analyzed the fracture healing time (6–10, 13–19, 21, 23, 27, 28) and reported 1,007 cases in total (467 in FNS and 540 in CCS). There was a strong heterogeneity among the studies (*I*^2 ^= 94% > 50%, *P *< 0.05). Heterogeneity persisted after sensitivity analysis and was analyzed using the random-effects model. Compared with the CCS group, the fracture healing time in the FNS group was shorter [MD = −0.75, 95% CI = (−1.04, −0.46)], and the difference was statistically significant (*P *< 0.05). Figure 2 shows the results.

#### Incidence of fracture non-union

**Figure 3:**
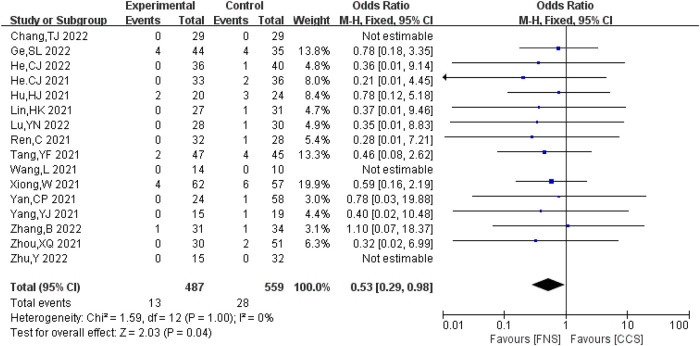
Forest plot comparisons for fracture non-union incidence.

A total of 16 studies (7–10, 13, 16–18, 20–23, 25, 26) analyzed the incidence of bone non-union. A total of 1,046 cases (487 in the FNS group and 559 in the CCS group) were reported, with no significant heterogeneity between the studies (*I*^2 ^= 0 < 50%, *P *> 0.05). The fixed-effects model was used for analysis. The incidence of bone non-union in the FNS group was lower compared with that in the CCS group [OR = 0.53, 95% CI = (0.29, 0.98)], and the difference was statistically significant (*P *< 0.05). Figure 3 shows the results.

#### Incidence of femoral head necrosis

**Figure 4:**
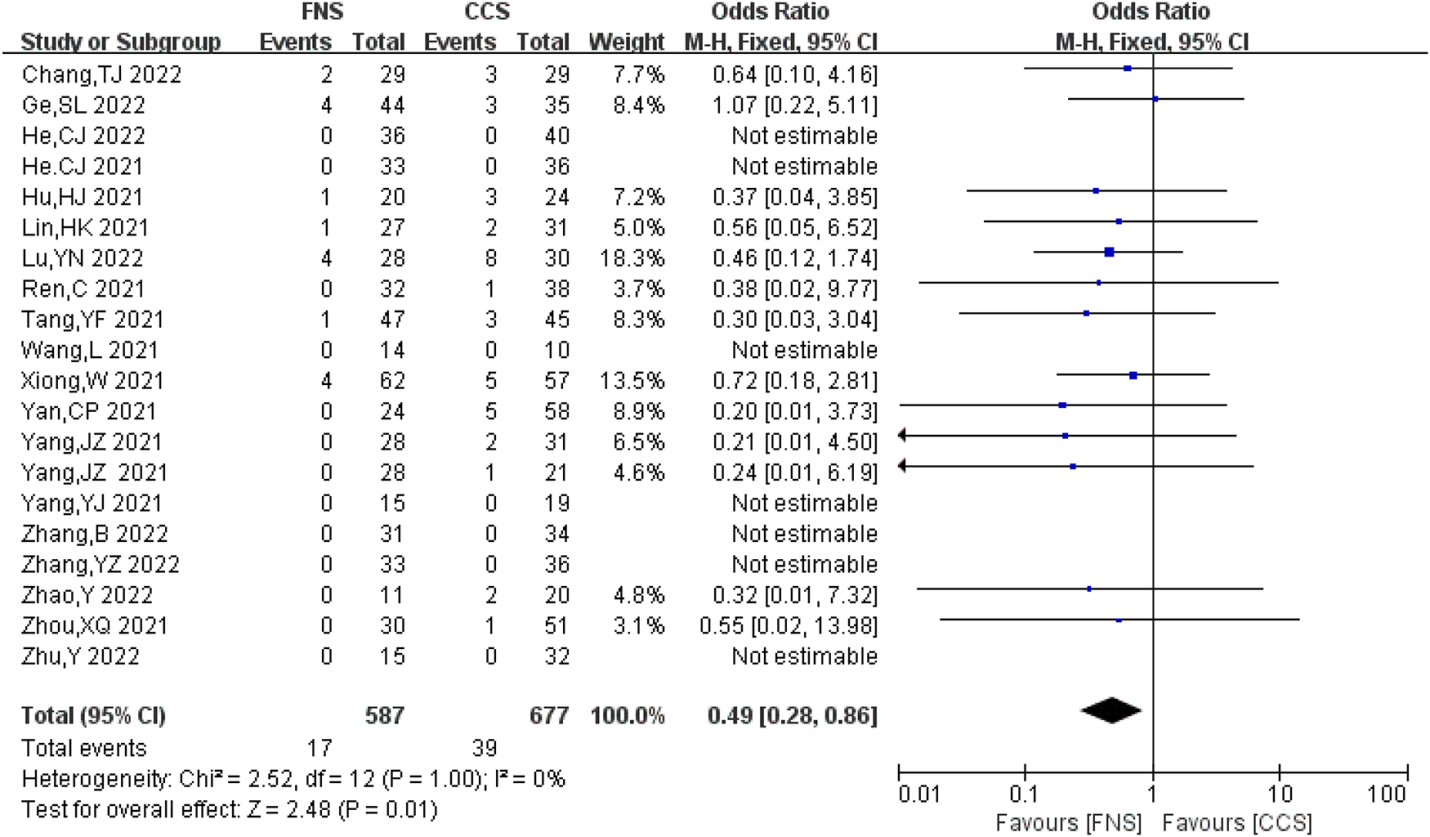
Forest plot comparisons for femoral head necrosis incidence.

A total of 20 studies (6–10, 13–24, 26, 28) analyzed the incidence of femoral head necrosis, involving 1,264 patients (587 in the FNS group and 677 in the CCS group), with no significant heterogeneity between studies (*I*^2^ = 0 < 50%, *P *> 0.05). The fixed-effects model was used for analysis. The incidence of osteonecrosis of the femoral head in the FNS group was lower compared with that in the CCS group [OR = 0.49, 95% CI = (0.28, 0.86)], and the difference was statistically significant (*P *< 0.05). Figure 4 shows the results.

#### Incidence of internal fixation failure

**Figure 5:**
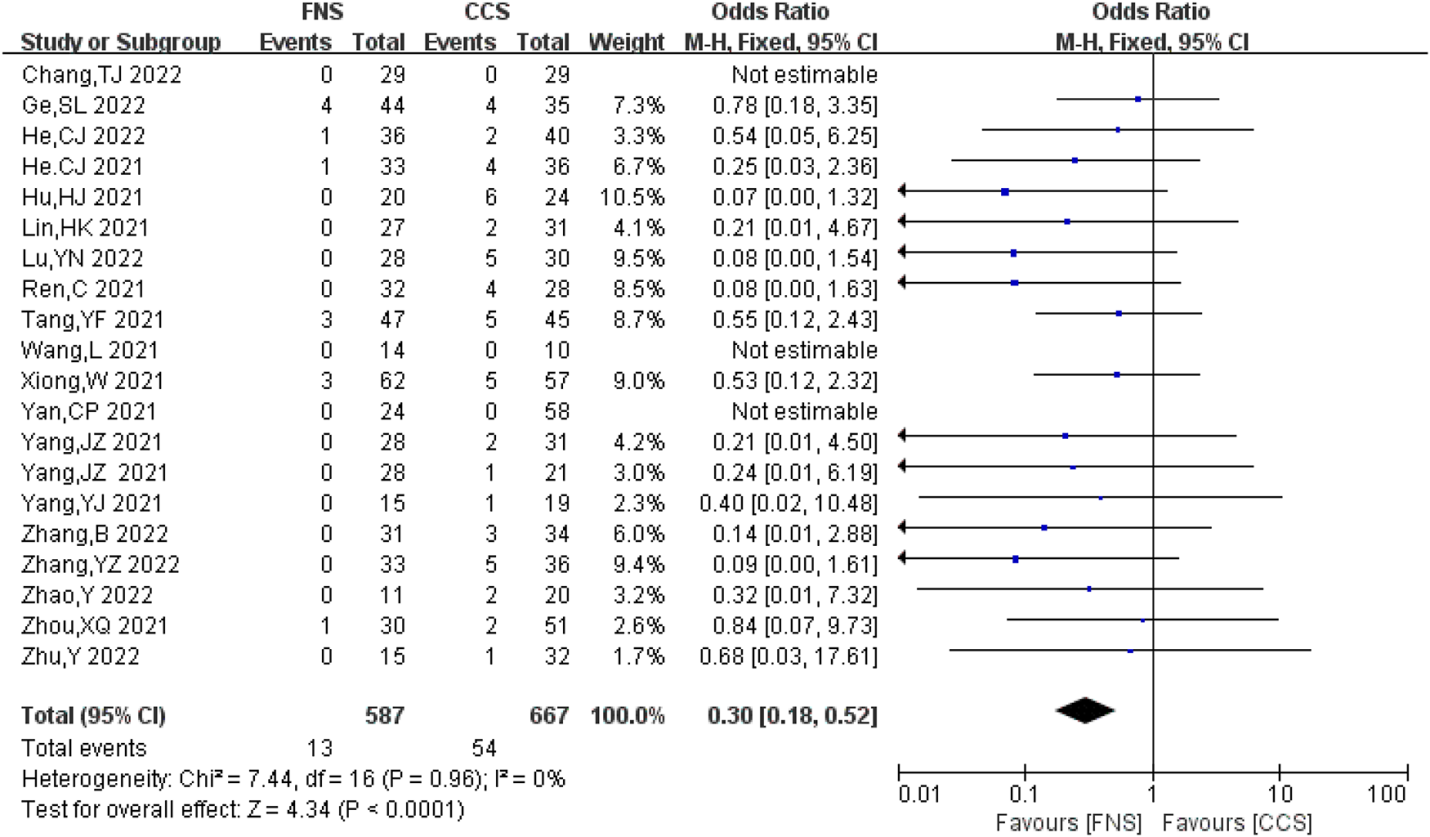
Forest plot comparisons for internal fixation failure incidence.

A total of 20 studies analyzed the incidence of internal fixation failure (6–10, 12–26), involving 1,254 patients in total (587 in FNS and 667 in CCS). There was no significant heterogeneity between the studies (*I*^2^ = 0 < 50%, *P *> 0.05), and the fixed-effects model was used for analysis. Compared with the CCS group, the FNS group had a lower rate of internal fixation failure [OR = 0.30, 95% CI = (0.18, 0.52)], and the difference was statistically significant (*P *< 0.05). Figure 5 shows the results.

#### Rate of femoral neck shortening

**Figure 6:**
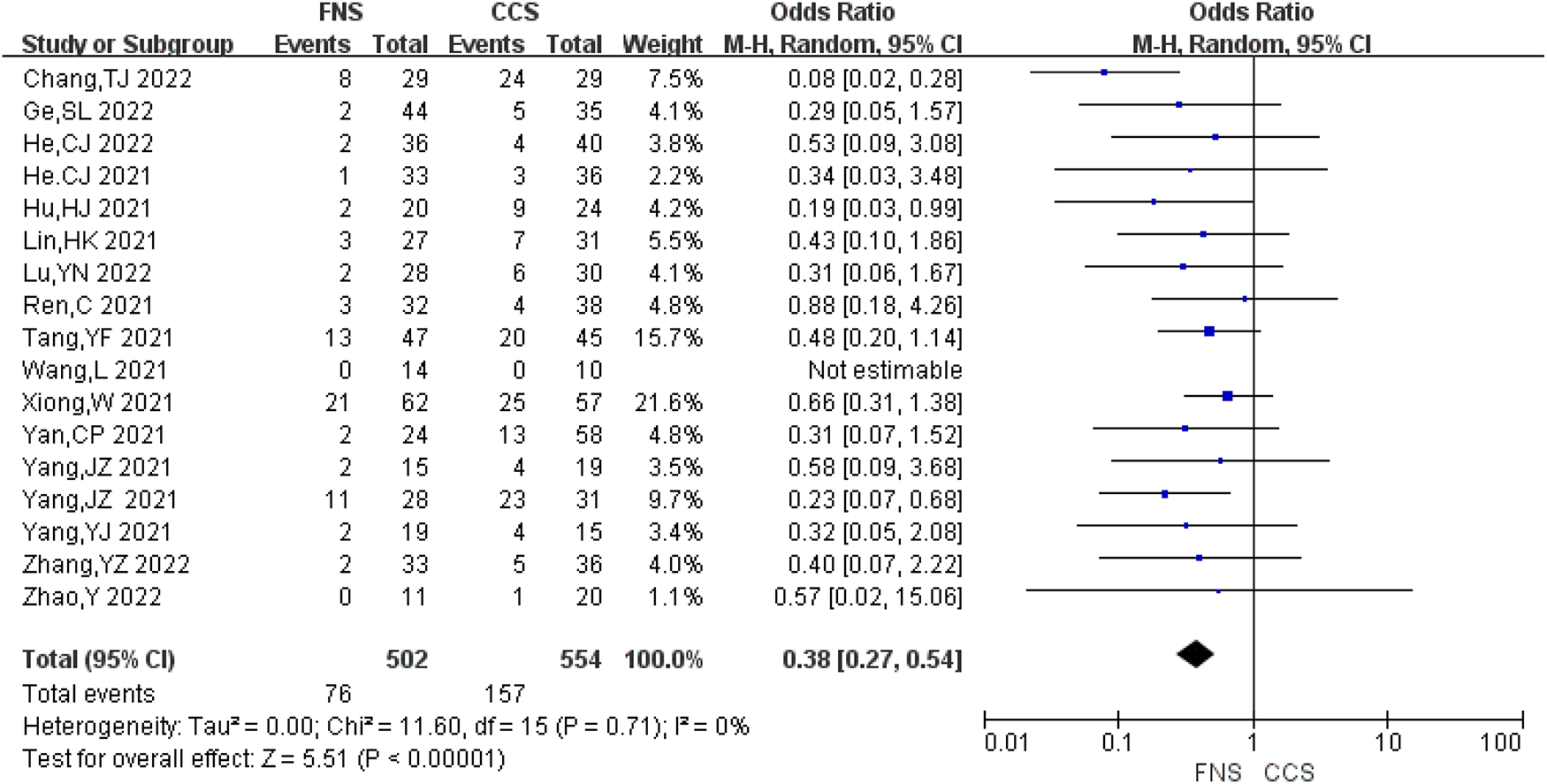
Forest plot comparisons for femoral neck shortening rate.

A total of 17 studies (6–10, 13–15, 17–23, 25) analyzed the rate of femoral neck shortening, involving 1,056 patients in total (502 in FNS and 554 in CCS), with no significant heterogeneity between studies (*I*^2^ = 0 < 50%, *P *> 0.05). The fixed-effects model was used for analysis. Compared with the CCS group, the FNS group had a lower rate of femoral neck shortening [OR = 0.38, 95% CI = (0.27, 0.54)], and the difference was statistically significant (*P *< 0.05). Figure 6 shows the results.

#### Harris hip score

**Figure 7:**
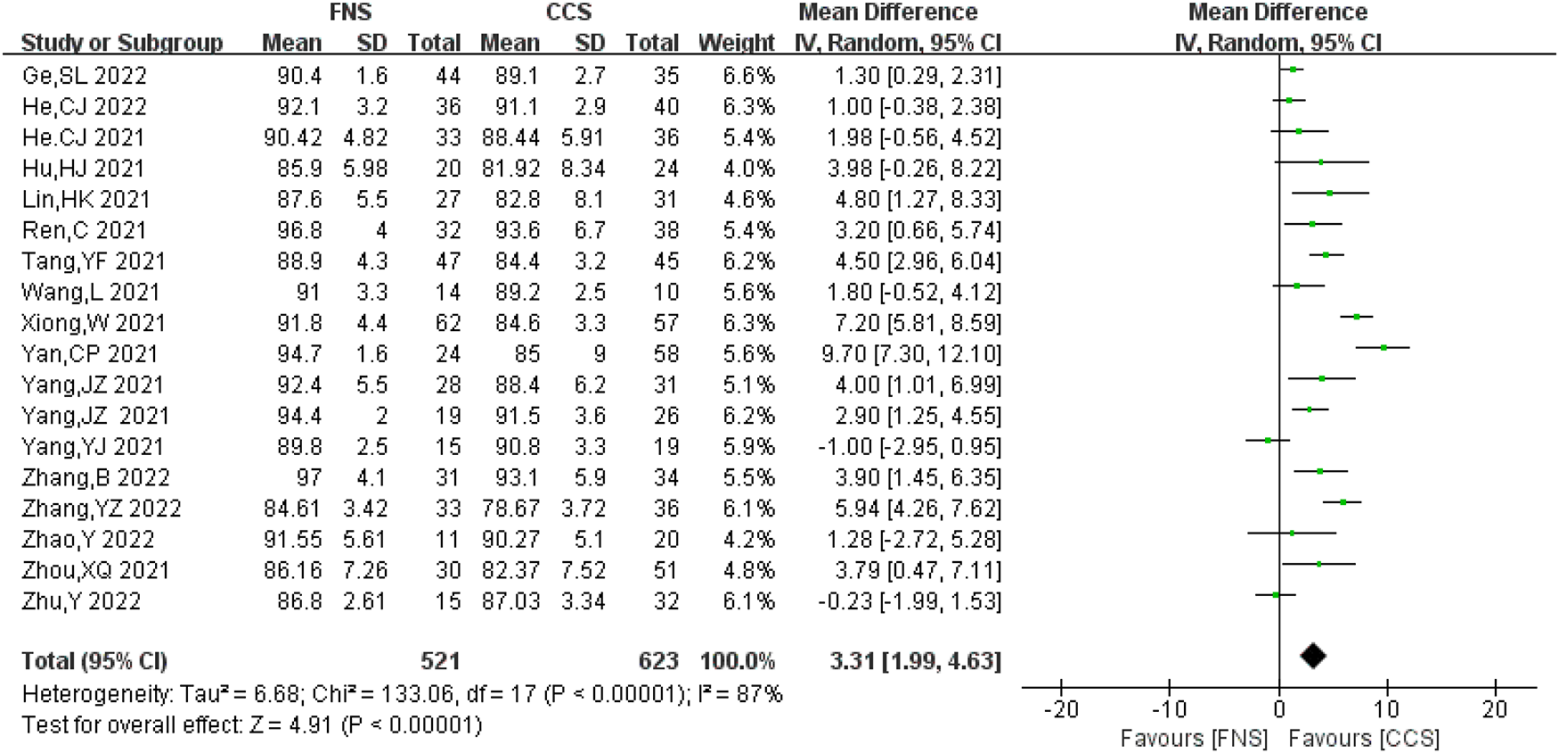
Forest plot comparisons for Harris hip score.

A total of 18 studies (6–9, 12–19, 21–26, 28) analyzed the Harris hip joint score, involving 1,144 patients in total (521 in the FNS group and 623 in the CCS group), with a strong heterogeneity between studies (*I*^2^ = 87% > 50%, *P *< 0.05). Heterogeneity persisted after sensitivity analysis, and the random-effects model was used for analysis. Hip function recovery in the FNS group was better compared with that in the CCS group [MD = 3.31, 95% CI = (1.99, 4.63)], with a statistically significant difference (*P *< 0.05). Figure 7 shows the results.

#### Barthel index

**Figure 8:**
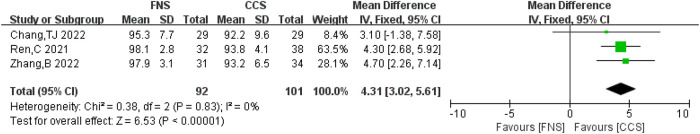
Forest plot comparisons for Barthel index.

A total of three studies (13, 20, 24) analyzed the Barthel index, involving 193 patients (92 in the FNS group and 101 in the CCS group). There was no significant heterogeneity between the studies (*I*^2 ^= 0 < 50%, *P *> 0.05), and the fixed-effects model was used for analysis. Compared with the CCS group, the FNS group recovered better in basic activities of daily living [OR = 4.31, 95% CI = (3.02, 5.61)], and the difference was statistically significant (*P *< 0.05). Figure 8 shows the results.

#### Operation time

**Figure 9:**
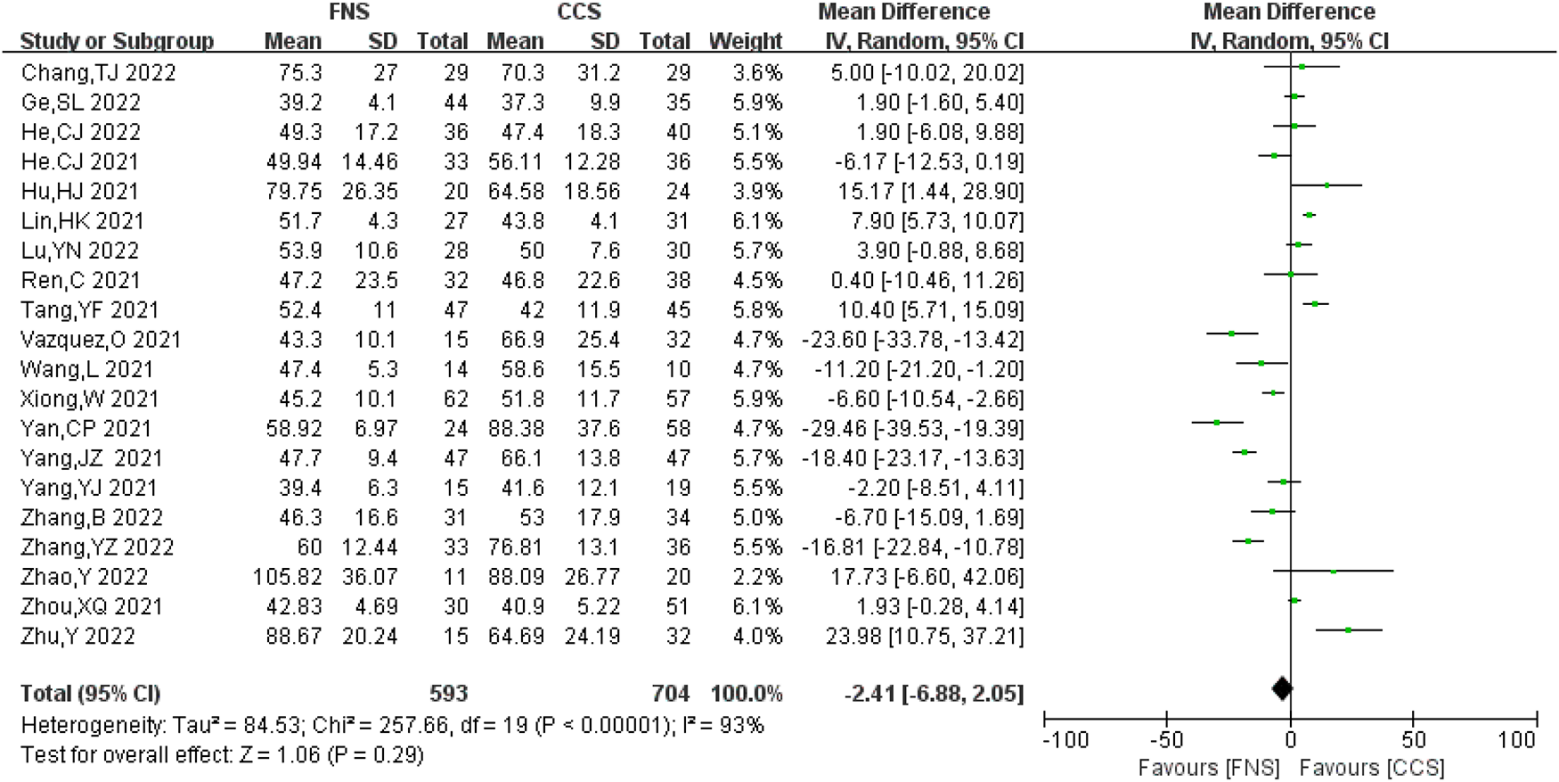
Forest plot comparisons for operation time.

A total of 20 studies (6–21, 23–26) analyzed the operation time, involving 1,297 patients in total (593 in the FNS group and 704 in the CCS group), with significant heterogeneity between studies (*I*^2 ^= 93% > 50%, *P *< 0.05). Heterogeneity persisted after sensitivity analysis, and the random-effects model was used for analysis. Compared with the CCS group, the FNS group had no significant difference in the operation time [MD = −2.41, 95% CI = (−6.88, 2.05)], with no significant difference (*P *> 0.05). Figure 9 shows the results.

#### Intraoperative blood loss

**Figure 10:**
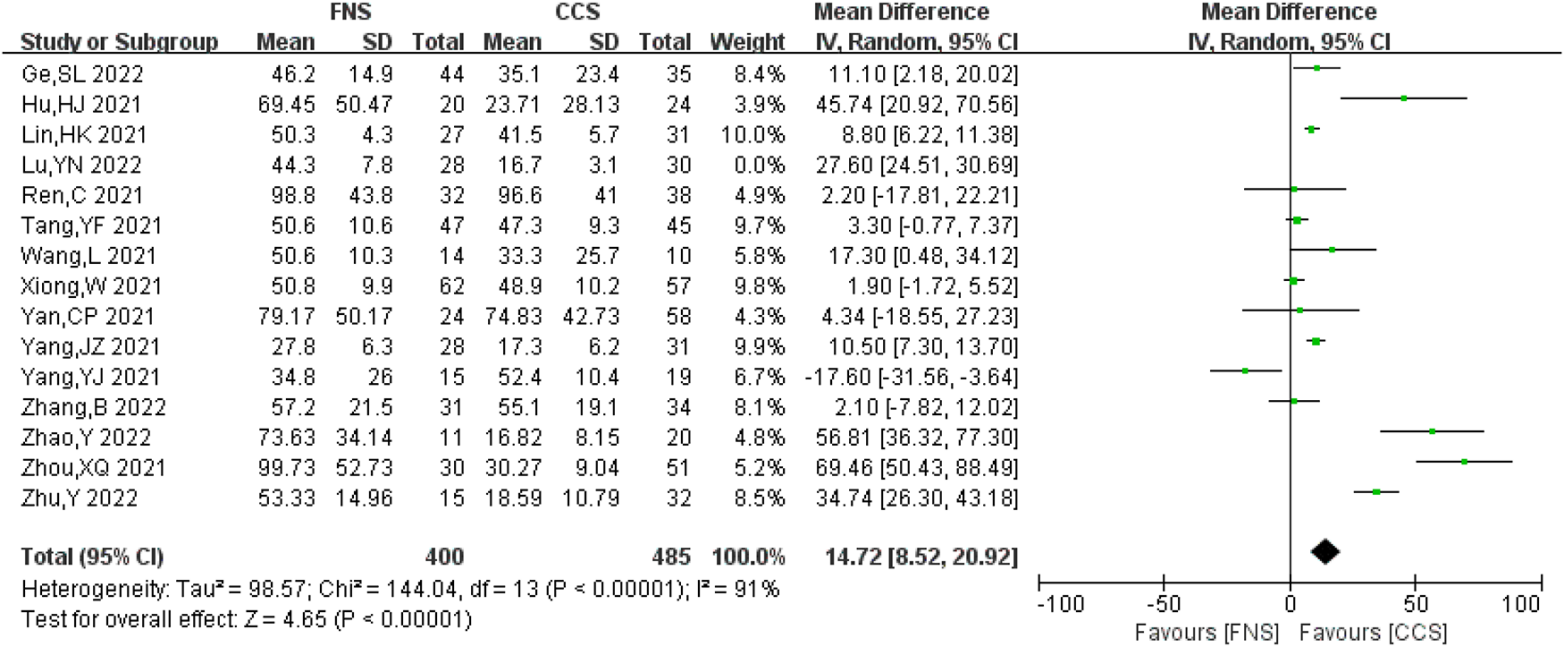
Forest plot comparisons for intraoperative blood loss.

A total of 15 studies (6, 8–10, 13, 15–19, 22–26) analyzed intraoperative blood loss, including 885 patients (400 in the FNS group and 485 in the CCS group). There was a strong heterogeneity between the studies (*I*^2 ^= 91%>50%, *P *< 0.05). Heterogeneity persisted after sensitivity analysis, and the random-effects model was used for analysis. Compared with the CCS group, the FNS group had more blood loss [MD = 14.72, 95% CI = (8.52, 20.92)], and the difference was statistically significant (*P *< 0.05). Figure 10 shows the results.

#### Fluoroscopy frequency

**Figure 11:**
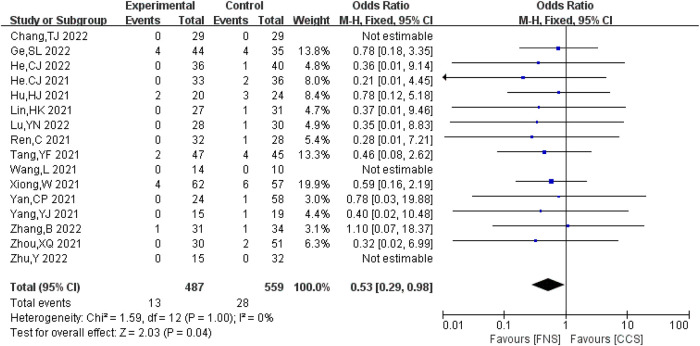
Forest plot comparisons for fluoroscopy frequency.

A total of 16 studies (6–10, 13, 16–18, 20–26) analyzed fluoroscopy frequency and reported a total of 1,046 cases (487 in FNS and 559 in CCS), with no significant heterogeneity between the studies (*I*^2 ^= 0 < 50%, *P *> 0.05). The fixed-effects model was used for analysis. Compared with the CCS group, the FNS group had a lower fluoroscopy frequency [OR = 0.53, 95% CI = (0.29, 0.98)], and the difference was statistically significant (*P *< 0.05). Figure 11 shows the results.

#### Complications

**Figure 12:**
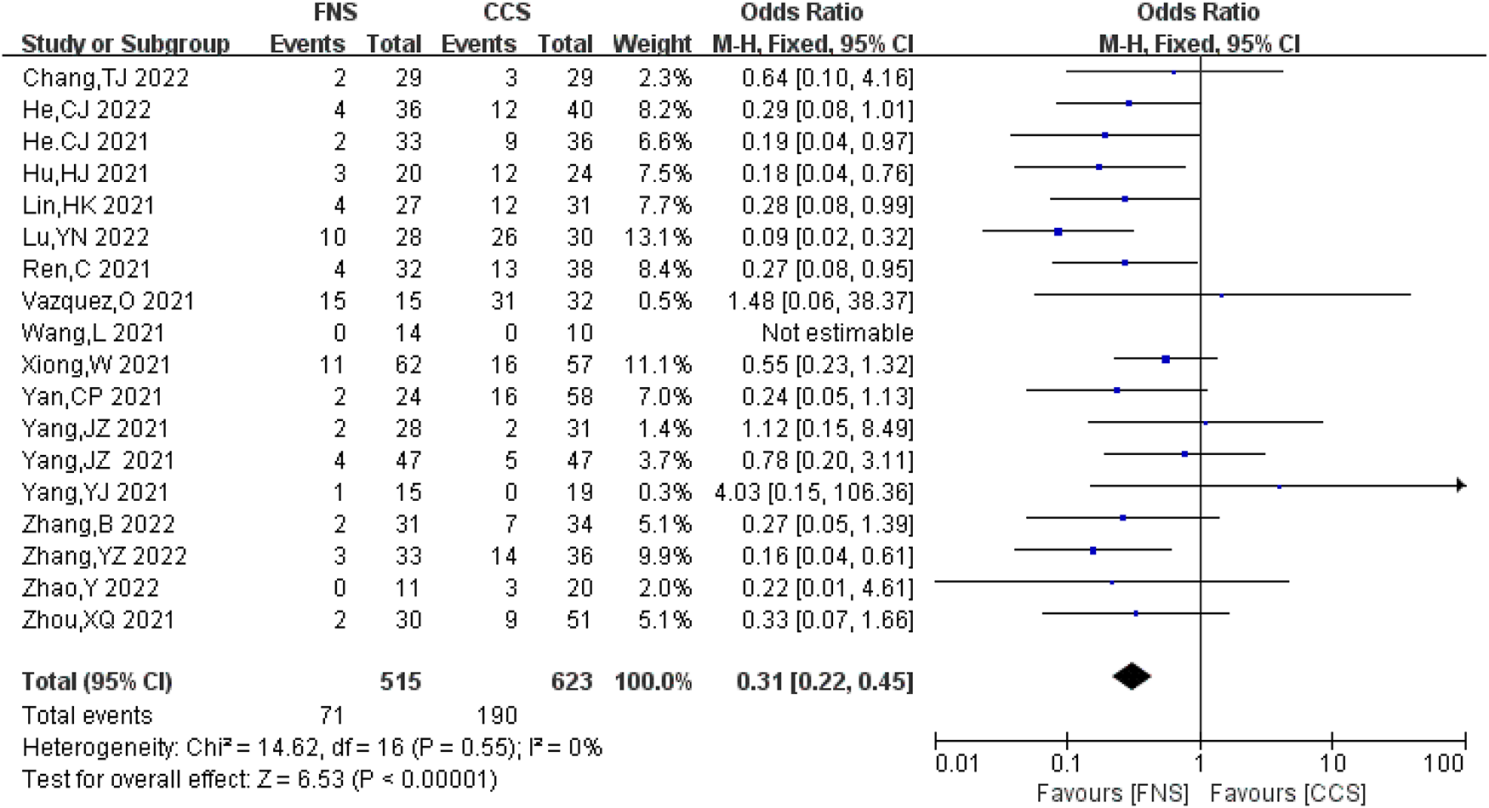
Forest plot comparisons for complications.

A total of 18 studies (6, 7, 9–15, 17–22, 24–26) analyzed complications involving 1,138 patients (515 in the FNS group and 623 in the CCS group). There was no significant heterogeneity between the studies (*I*^2^ = 0 < 50%, *P *> 0.05), and the fixed-effects model was used. The probability of complications in the FNS group was significantly lower compared with that in the CCS group [OR = 0.31, 95% CI = (0.22, 0.45)], and the difference was statistically significant (*P *< 0.05). Figure 12 shows the results.

### Publishing bias test

**Figure 13:**
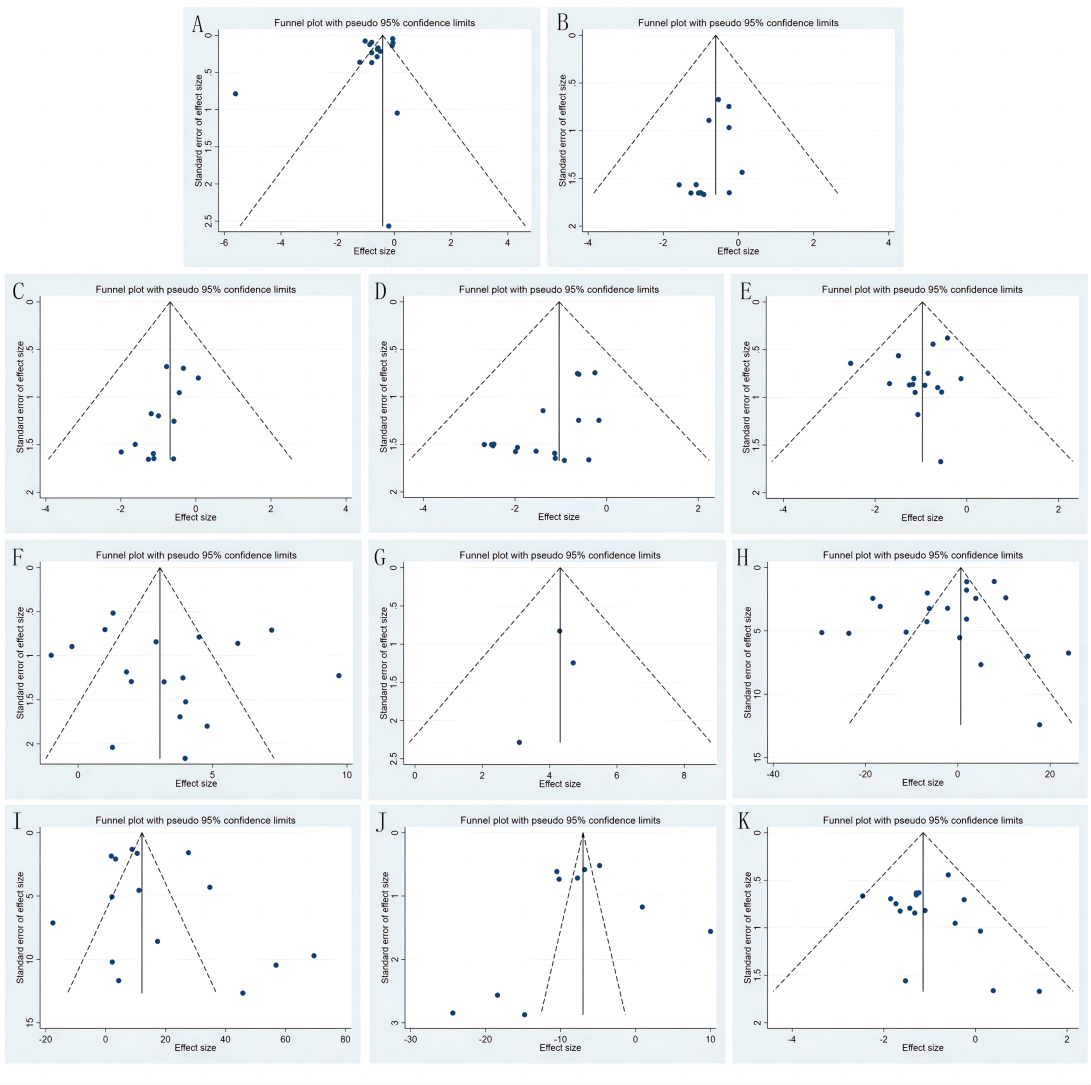
Funnel charts for healing time (**A**), fracture non-union incidence (**B**), femoral head necrosis incidence (**C**), internal fixation failure incidence (**D**), femoral neck shortening rate (**E**), Harris hip score (**F**), Barthel index (**G**), operation time (**H**), intraoperative blood loss (**I**), fluoroscopy frequency (**J**), and complications (**K**).

The funnel chart (Figure 13) and Egger's test (Table 3) for all outcomes found publication bias for the rate of osteonecrosis of the femoral head (*P *= 0.013) and the rate of internal fixation failure (*P *= 0.004) and no publication bias for the remaining included measures (Egger's test, *P *> 0.05). The above publication bias could be attributed to the fact that databases other than Chinese and English were not searched, and the negative results have not been published.

**Table 3:** Egger's test of publication bias.

Outcome	SE	LL	UL	*P*
Healing time	1.427	−5.56	0.562	0.102
Fracture non-union incidence	0.254	−1.086	0.032	0.062
Femoral head necrosis incidence	0.319	−1.641	−0.237	0.013
Internal fixation failure incidence	0.412	−2.276	−0.518	0.004
Femoral neck shortening rate	0.639	−1.87	0.871	0.448
Harris hip score	1.829	−2.696	5.058	0.528
Barthel index	0.783	−10.549	9.342	0.582
Operation time	1.448	−5.184	0.9	0.156
Intraoperative blood loss	1.839	−2.721	5.223	0.508
Complications	0.762	−0.925	2.322	0.374
Fluoroscopy frequency	4.009	−10.17	8.32	0.823

LL and UL represent the lower and upper limits of the 95% confidence interval of Egger's regression intercept.

### Results of sensitivity analyses

**Figure 14:**
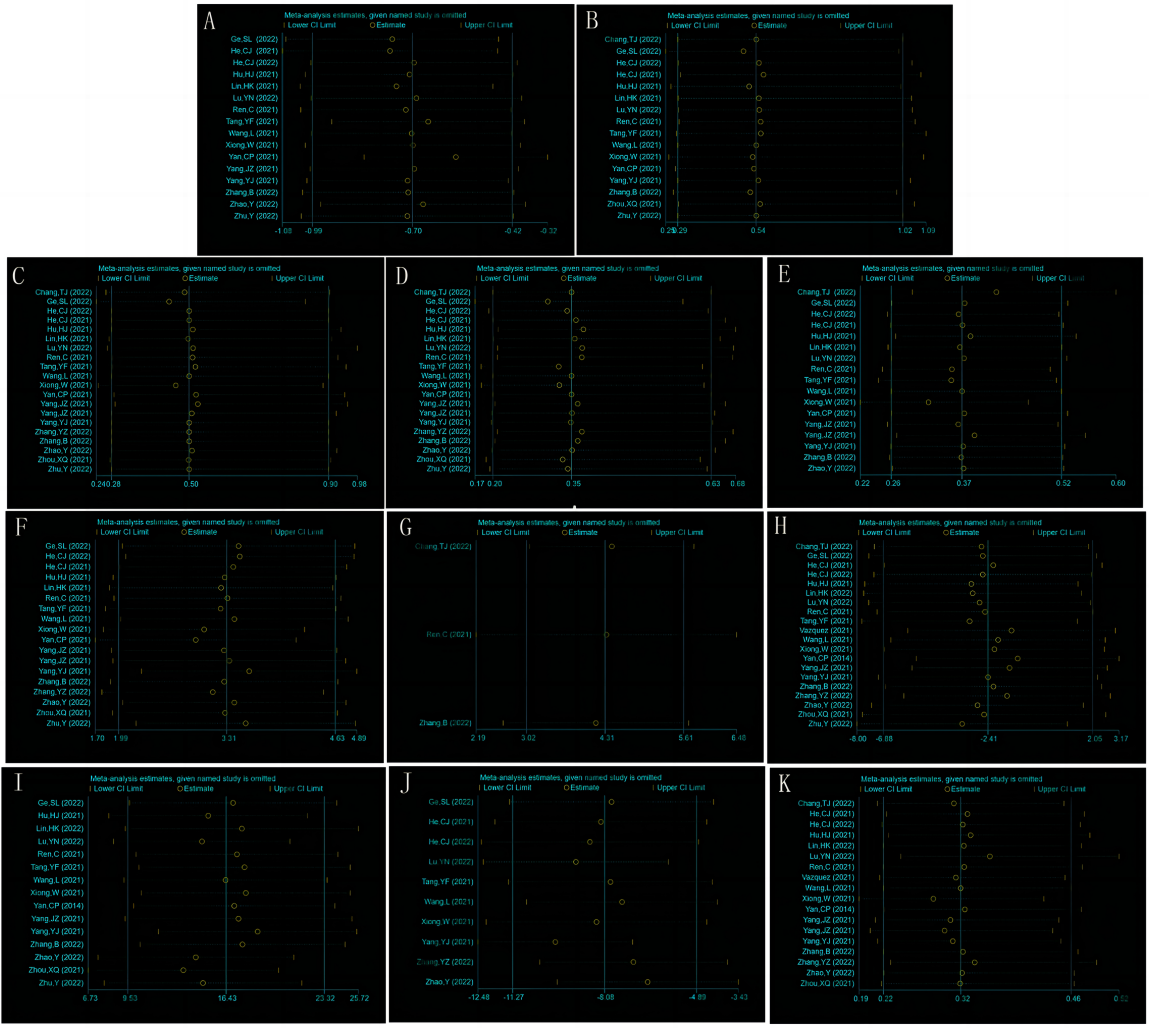
Sensitivity analyses for healing time (**A**), fracture non-union incidence (**B**), femoral head necrosis incidence (**C**), internal fixation failure incidence (**D**), femoral neck shortening rate (**E**), Harris hip score (**F**), Barthel index (**G**), operation time (**H**), intraoperative blood loss (**I**), fluoroscopy frequency (**J**), and complications (**K**).

Sensitivity analyses were performed using Stata v14.0 for all the outcomes. Pooled analysis of each study after exclusion resulted in no qualitative change in meta-analysis results, suggesting that all outcome indicators were relatively robust (Figure 14).

## Discussion

In this study, 21 retrospective studies published between 2021 and 2022 were included, of which 20 were conducted in China and 1 was in Switzerland, involving a total of 1,347 patients. There were 612 cases in the FNS group and 735 cases in the CCS group. The results showed that compared with CCS, FNS had better effects on femoral neck fractures, with shorter fracture healing time, lower incidence of bone non-union, lower incidence of femoral head necrosis, lower incidence of internal fixation failure, better hip function, better basic activities of daily living, and fewer complications. In addition, compared with the operation of CCS, the operation of FNS had less fluoroscopy frequency and more bleeding during operations. Nevertheless, there is no difference in operation time between the two operations. In a word, these results provided support for FNS as an effective and safe internal fixation for patients with femoral neck fractures. This study is a meta-analysis including most literature and patients. Therefore, the results of this meta-analysis are credible, with a high level of evidence to provide a theoretical basis for the use of FNS in treating femoral neck fractures.

With regard to femoral neck fractures, fracture non-union and femoral head necrosis are the most common problems because of damaged blood supply of the femoral head, including the retinacular artery, nutrient artery of the femoral shaft, and foveal artery, during trauma (29, 30). As shown in this meta-analysis, 16 studies, including 1,007 patients, reported the bone healing time of FNS and CCS separately, indicating that the bone healing time of patients subjected to FNS is significantly shorter than that of patients subjected to CCS, with high heterogeneity. Although we performed a sensitivity analysis, heterogeneity is still high. The possible reasons are related to different time units (e.g., months and weeks), different ages of patients, and different types of femoral neck fractures included in different studies. In addition, 16 studies with 1,046 patients and 20 studies with 1,264 patients reported the incidence of fracture non-union and femoral head necrosis separately. The results of the meta-analysis show that the incidence of fracture non-union and avascular necrosis of patients treated by FNS is lower than that of patients treated by CCS, with low heterogeneity. The reasons for the above results may be related to the effects of anti-rotation, dynamic compression, and locking mechanisms provided by FNS. According to biomechanical studies (31–33), the mechanical stability and rotational stability of FNS are both better than those of CCS, and the effects of anti-rotation, dynamic compression, or mechanical stability and rotational stability are beneficial to both bone healing and blood supply of the femoral head. In addition, the small size and minimally invasive operation of FNS are other reasons for a lower incidence of femoral head necrosis after FNS internal fixation compared with CCS internal fixation. However, more RCT with long follow-up times are needed to further verify these results.

The incidence of internal fixation failure and femoral neck shortening can negatively affect hip function, which is important in evaluating the clinical effects and safety of internal fixation. According to statistics (34), approximately half of the patients with femoral neck fractures have femoral neck shortening of more than 5 mm. Because femoral neck fractures, especially Pauwels type III fractures, are extremely unstable with high shear force and shear stress, they are prone to internal fixation failure and fracture fragment displacement. Although CCS internal fixation has the abilities of compression and rotation resistance, it may cause a high risk of femoral neck shortening, coxa varus, and internal fixation failure for femoral neck fractures (35). In this meta-analysis, the incidences of internal fixation failure and femoral neck shortening of FNS are lower than those of CCS, notably with low heterogeneity, consistent with previous studies. Some biomechanical studies (36–38) have reported that FNS has better mechanical stability than CCS, including less stress concentration, less stress shielding, and smaller peak displacement of the femoral head. In addition, the contact pressure between fracture fragments caused by FNS is smaller than that of CCS, which may be the reason for a lower incidence of femoral neck shortening caused by FNS (9).

In order to compare the surgical trauma caused by FNS and CCS during operation for patients with femoral neck fractures, the present study analyzed the operation time, intraoperative blood loss, and fluoroscopy frequency of FNS and CCS. The results showed that compared with CCS, FNS had more intraoperative blood loss, and the fluoroscopy frequency of FNS was less. Meanwhile, there is no difference in operation time between FNS and CCS. The heterogeneity may originate from the surgical skill of surgeons and different types of femoral neck fractures. The increased intraoperative blood loss associated with FNS may be due to the incision made on the proximal femur and the cutting of the vastus lateralis muscle to expose the proximal femoral shaft at the level of the lesser trochanter to place the FNS plate, causing hemorrhage. The reduced fluoroscopy frequency of FNS may be attributed to the utilization of guide equipment during FNS operation, which is helpful in decreasing fluoroscopy frequency. In contrast, it is hard to insert CCS in an inverted triangle with lower fluoroscopy frequency (39).

In terms of complications, 18 studies with 1,138 patients were included in the meta-analysis, and the result showed that there were fewer complications caused by FNS compared with CCS, with low heterogeneity, indicating that FNS is a safe internal fixation for patients with femoral neck fractures (26).

The Harris hip joint score is an important outcome in evaluating the clinical effect of internal fixation for femoral neck fractures, which is related to some factors such as bone healing, osteonecrosis of the femoral head, internal fixation failure, femoral neck shorting, coxa varus, surgical trauma, and complications, and the higher Harris hip joint score, the better the hip function (40). Not surprisingly, the result of the meta-analysis shows that the Harris hip joint score of patients treated by FNS, who had short bone healing time and low incidence of fracture non-union, osteonecrosis of the femoral head, internal fixation failure, femoral neck shorting, minor surgical trauma, and fewer complications, is higher than that of patients treated by CCS, with high heterogeneity, which may be related to the subjectivity of evaluators. Meanwhile, the Barthel index of patients treated by FNS is better than that of patients treated by CCS, indicating that the activities of daily living of patients treated by FNS are better than those of patients treated by CCS. Both Harris hip score and Barthel index showed that patients with femoral neck fractures receiving FNS treatment recovered better than those treated with CCS, and FNS is an effective internal fixation for femoral neck fractures.

Although this meta-analysis has some advantages, including literature with more than 1,000 patients and analysis of more outcomes related to clinical effects and safety of FNS and CCS, there also are some limitations: (i) Most studies included are from China, where there are many cases of femoral neck fractures. (ii) All studies included are retrospective studies with short follow-up time, which is not enough to evaluate the effects and safety of internal fixations on the viewpoint of evidence-based medicine. (iii) Operation delay is considered a risk factor for necrosis after internal fixation. However, this indicator was not analyzed in this article due to the inconsistency in the division of the time interval between injury and surgery in the original studies. (iv) Partial results have high heterogeneity and publishing bias. Thus, many large-sample, multi-center RCT with long follow-up times are needed to verify the clinical effects and safety of FNS on femoral neck fractures.

In a word, although there are some limitations in this meta-analysis, the conclusion of the study that FNS, an effective and safe internal fixation for femoral neck fractures, is better than CCS is credible in our opinion.

## Data Availability

The raw data supporting the conclusions of this article will be made available by the authors, without undue reservation.

## Supplementary material

**SUPPLEMENTARY TABLE S1**

The search strategy and results of PubMed.

**SUPPLEMENTARY TABLE S2**

The search strategy and results of Embase.

**SUPPLEMENTARY TABLE S3**

The search strategy and results of the Cochrane Library.

**SUPPLEMENTARY FIGURE S1**

Egger’s test for healing time (A), fracture non-union incidence (B), femoral head necrosis incidence (C), internal fixation failure incidence (D), femoral neck shortening rate (E), Harris hip score (F), Barthel index (G), operation time (H), intraoperative blood loss (I), fluoroscopy frequency (J), and complications (K).
