# Supplementary material for: Femoral neck system vs. cannulated screws on treating femoral neck fracture: a meta-analysis and system review
Source: Front Surg. 2023 Jul 18;10:1224559. doi: 10.3389/fsurg.2023.1224559 (PMC10390772; doi:10.3389/fsurg.2023.1224559)
Supplement: Supplementary file 1 [file Datasheet1.docx]

**Supplementary Material**

**Table S1.** The search strategy and results of PubMed

| Search | Query | Items found |
| --- | --- | --- |
| #1 | ((((Femoral Neck Fractures [MeSH Terms]) OR (Femoral Neck Fracture)) OR (Femur Neck Fractures)) OR (Femur Neck Fracture)) OR (intracapsular hip fracture) | 19633 |
| #2 | (Femoral neck system) OR (FNS) | 4025 |
| #3 | (((((Femoral Neck Fractures [MeSH Terms]) OR (Femoral Neck Fracture)) OR (Femur Neck Fractures)) OR (Femur Neck Fracture)) OR (intracapsular hip fracture)) AND ((Femoral neck system) OR (FNS)) | 1390 |
| #4 | (((((bone screws [MeSH Terms]) OR (Cannulated cancellous screws)) OR (Cannulated screws)) OR (Cannulated compression screws)) OR (CCS)) OR (CS) | 558345 |
| #5 | ((((((Femoral Neck Fractures [MeSH Terms]) OR (Femoral Neck Fracture)) OR (Femur Neck Fractures)) OR (Femur Neck Fracture)) OR (intracapsular hip fracture)) AND ((Femoral neck system) OR (FNS))) AND ((((((bone screws [MeSH Terms]) OR (Cannulated cancellous screws)) OR (Cannulated screws)) OR (Cannulated compression screws)) OR (CCS)) OR (CS)) | 228 |

**Table S2.** The search strategy and results of Embase

| Search | Query | Items found |
| --- | --- | --- |
| #1 | 'femoral neck fracture'/exp | 14112 |
| #2 | 'femur neck fracture': ab,ti | 131 |
| #3 | 'femur neck fractures': ab,ti | 128 |
| #4 | 'hip fracture': ab,ti | 22045 |
| #5 | #1 OR #2 OR #3 OR #4 | 34513 |
| #6 | 'femoral neck system'/exp | 16 |
| #7 | 'femoral neck system': ab,ti | 48 |
| #8 | 'fns': ab,ti | 1340 |
| #9 | #6 OR #7 OR #8 | 1354 |
| #10 | 'cannulated screw'/exp | 1686 |
| #11 | 'cancellous screw': ab,ti | 309 |
| #13 | 'cannulated screw': ab,ti | 896 |
| #15 | 'cannulated cancellous screw': ab,ti | 49 |
| #16 | 'compression screw': ab,ti | 733 |
| #17 | #12 OR #13 OR #14 OR #15 OR #16 | 3204 |
| #18 | #5 AND #9 AND #17 | 30 |

**Table S3**. The search strategy and results of the cochrane library

| Search | Query | Items found |
| --- | --- | --- |
| #1 | MeSH descriptor: [Femoral Neck Fractures] explode all trees | 472 |
| #2 | (Femur Neck Fractures): ti,ab,kw | 1251 |
| #3 | (Femoral Neck Fracture): ti,ab,kw | 2554 |
| #4 | (intracapsular hip fracture): ti,ab,kw | 148 |
| #5 | #1 OR #2 OR #3 OR #4 | 2922 |
| #6 | MeSH descriptor: [Bone Screws] explode all trees | 885 |
| #7 | (Cannulated cancellous screws): ti,ab,kw | 12 |
| #8 | (Cannulated screws): ti,ab,kw | 183 |
| #9 | (compression screws): ti,ab,kw | 363 |
| #10 | #6 OR #7 OR #8 OR #9 | 1270 |
| #11 | (Femoral neck system): ti,ab,kw | 429 |
| #12 | (FNS): ti,ab,kw | 82 |
| #13 | #11 OR #12 | 502 |
| #14 | #5 AND #10 AND #13 | 28 |
| #15 | Type a search term or use the S or MeSH buttons to compose | N/A |

**
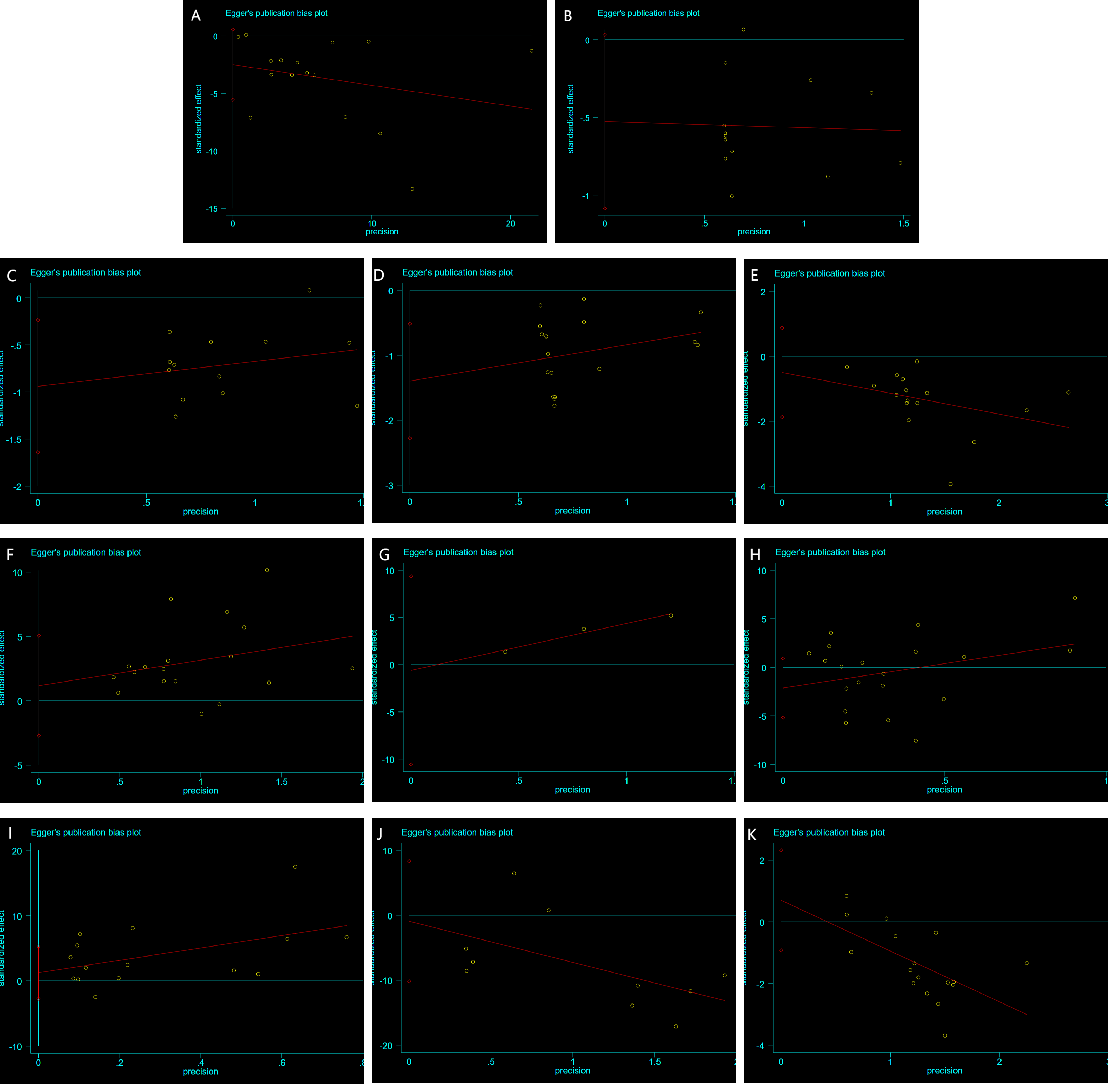
**

**Fig. S1** Egger’s test for Healing time (A), Fracture nonunion incidence (B), and Femoral head necrosis incidence (C), Internal fixation failure incidence (D), Femoral neck shortening rate (E), Harris hip score (F), Barthel index (G), Operation time (H), Intraoperative bleeding loss (I), Fluoroscopy frequency (J), Complications (K)
